# Top-down mechanisms of anesthetic-induced unconsciousness

**DOI:** 10.3389/fnsys.2014.00115

**Published:** 2014-06-23

**Authors:** George A. Mashour

**Affiliations:** Neuroscience Graduate Program, Department of Anesthesiology, Center for Consciousness Science, University of Michigan Medical SchoolAnn Arbor, MI, USA

**Keywords:** consciousness, anesthesia, anesthetic mechanisms, ketamine, propofol, sleep

## Abstract

The question of how structurally and pharmacologically diverse general anesthetics disrupt consciousness has persisted since the nineteenth century. There has traditionally been a significant focus on “bottom-up” mechanisms of anesthetic action, in terms of sensory processing, arousal systems, and structural scales. However, recent evidence suggests that the neural mechanisms of anesthetic-induced unconsciousness may involve a “top-down” process, which parallels current perspectives on the neurobiology of conscious experience itself. This article considers various arguments for top-down mechanisms of anesthetic-induced unconsciousness, with a focus on sensory processing and sleep-wake networks. Furthermore, recent theoretical work is discussed to highlight the possibility that top-down explanations may be causally sufficient, even assuming critical bottom-up events.

## Introduction and terminology

The mechanism by which structurally and pharmacologically diverse general anesthetics can render an individual unconscious has remained incompletely understood since 1846. One of the current controversies in the systems neuroscience approach to this question relates to the primacy of top-down vs. bottom-up mechanisms of anesthetic-induced unconsciousness. In the context of this article, the term “bottom-up” has three different meanings, which will always be explicit. The first meaning of “bottom-up” relates to a neurocognitive hierarchy of *sensory processing*, which might be best illustrated by considering the neural processing involved in visual consciousness. After retinal stimulation, visual information is transmitted to the lateral geniculate nucleus in the thalamus followed by transmission to the primary visual cortex (V1) in the occipital lobe. Visual processing thereafter follows two “streams” that flow dorsally to the prefrontal cortex and ventrally to the temporal lobe. Thus, visual information is received by peripheral sensors and transmitted in a bottom-up way to the thalamus, primary sensory cortex, higher modal processing areas, and multimodal association cortex. The second meaning of “bottom-up” relates to *arousal pathways*. From the pons to the midbrain to diencephalic structures such as the hypothalamus, a variety of subcortical nuclei project to and arouse the cortex through the actions of distinct neurotransmitters. This represents a bottom-up pathway from subcortical wake-promoting nuclei to the cortex, sometimes with a synaptic relay in the thalamus. The third meaning of “bottom-up” relates to processes that occur from smaller to larger structural scales, e.g., from the molecular, to the cellular, to the neuroanatomical, to the network level of the brain. These three meanings of “bottom-up”—referring to *sensory, arousal*, and *structural* hierarchies—all have relevance to understanding the mechanism of anesthetic-induced unconsciousness.

In the context of this article, the term “anesthetic-induced unconsciousness” refers to an unconscious cognitive state that is just below the threshold of normal conscious perception of the environment, with the further stipulation that the subject is not being exposed to a noxious stimulus (e.g., surgery). As an example, consider a human volunteer receiving an infusion of the intravenous anesthetic propofol that is titrated to higher concentrations until she can no longer follow verbal commands—this loss of responsiveness would be used as the (admittedly imperfect) surrogate of anesthetic-induced unconsciousness. It should be noted clearly that anesthetic-induced unconsciousness is not equivalent to surgical anesthesia, because a noxious stimulus such as a scalpel cutting through skin could easily reverse the unconscious state in our hypothetical volunteer. Anesthetic-induced unconsciousness may not be sufficient for surgical intervention and is therefore not necessarily equivalent to our clinical conception of general anesthesia.

## Why bottom-up frameworks of anesthetic-induced unconsciousness have been dominant

There are several reasons why bottom-up approaches to anesthetic-induced unconsciousness have dominated the field. With respect to the sensory or arousal hierarchy, consciousness has often been deconstructed into wakefulness (an active cortex, open eyes) and awareness (subjective experience). Our understanding of wakefulness and brain arousal as mediated by subcortical structures like the ascending reticular activating system dates back some 65 years to the work of Moruzzi and Magoun (1949). Similarly, the association of consciousness with processing at the level of the primary sensory cortex remained dominant until some 20 years ago when Crick and Koch formally questioned whether visual consciousness was correlated with activity in V1 (Crick and Koch, 1995). For more than a decade, the “thalamic switch” hypothesis of anesthetic-induced unconsciousness—which is characterized by the blockade of sensory information from thalamus to primary sensory cortex—has been highly influential (Alkire et al., 2000). Thus, the longstanding viewpoint of consciousness and anesthesia as linked to subcortical arousal and primary sensory processes is significantly more entrenched than the relatively recent perspective of consciousness as a higher-order experiential process generated by network communication across association cortices. With respect to structural scales, there is a general scientific tendency to think of the term “mechanism” as a bottom-up process in which actions at the molecular or “micro” level lead to causal events that result in changes at the “macro” level. From this perspective, macro-level explanations merely supervene on micro-level explanations (Hoel et al., 2013). This is also true in the history of research into anesthetic-induced unconsciousness. Since 1847, there have been a series of micro-level approaches to anesthetic mechanism, including effects on lipids (1847 through the mid-1980s; for review of early theories see Perouansky, 2012) and proteins (mid-1980s until the present; Franks and Lieb, 1984). Thus, the targets of general anesthetics have traditionally been molecular, the foundational level of a bottom-up structural hierarchy.

The 1990s was an important era in anesthetic mechanisms research for four reasons. First, it became clear that there was a neuroanatomical segregation of the major therapeutic endpoints of general anesthesia, with immobility mediated in the spinal cord vs. unconsciousness and amnesia mediated in the brain (Antognini and Schwartz, 1993; Rampil, 1994). Second, the first neuroimaging studies of general anesthesia were conducted, pointing to regional differences in anesthetic action rather than global brain suppression (Alkire et al., 1995). Third, specific anesthetic binding sites on neurotransmitter receptors (most notably, the gamma-aminobutyric acid [GABA] receptor) were identified (Mihic et al., 1997). Fourth, it was first suggested that general anesthetics might suppress consciousness through actions on the subcortical nuclei that evolved to control sleep-wake states (Lydic and Biebuyck, 1994), a decidedly systems neuroscience approach. At least two of these developments—effects of anesthetics on neurotransmitter receptors and sleep-wake nuclei—are explicitly bottom-up approaches to anesthetic mechanisms. The focus on the molecular mediators of anesthetic action represents a bottom-up explanation from the perspective of structural scales and the “shared circuits” hypothesis of sleep and anesthesia represents a bottom-up explanation in terms of arousal systems. Both have received considerable attention and enjoy significant empirical support. The goal of the present article is not to argue that anesthetics have no effects on molecular targets or subcortical structures such as sleep-wake nuclei, but rather to counterbalance the tendency to think of anesthetic mechanisms as a fundamentally or exclusively bottom-up process. There are now several compelling lines of evidence to suggest that consciousness and anesthetic-induced unconsciousness in humans are higher-order processes and that, in principle, macro-level mechanisms can be causally sufficient to explain such emergent phenomena.

## Arguments supporting top-down mechanisms of anesthetic-induced unconsciousness

### Consciousness and anesthetic-induced unconsciousness are associated with multimodal association cortex rather than primary sensory cortex

**Figure 1:**
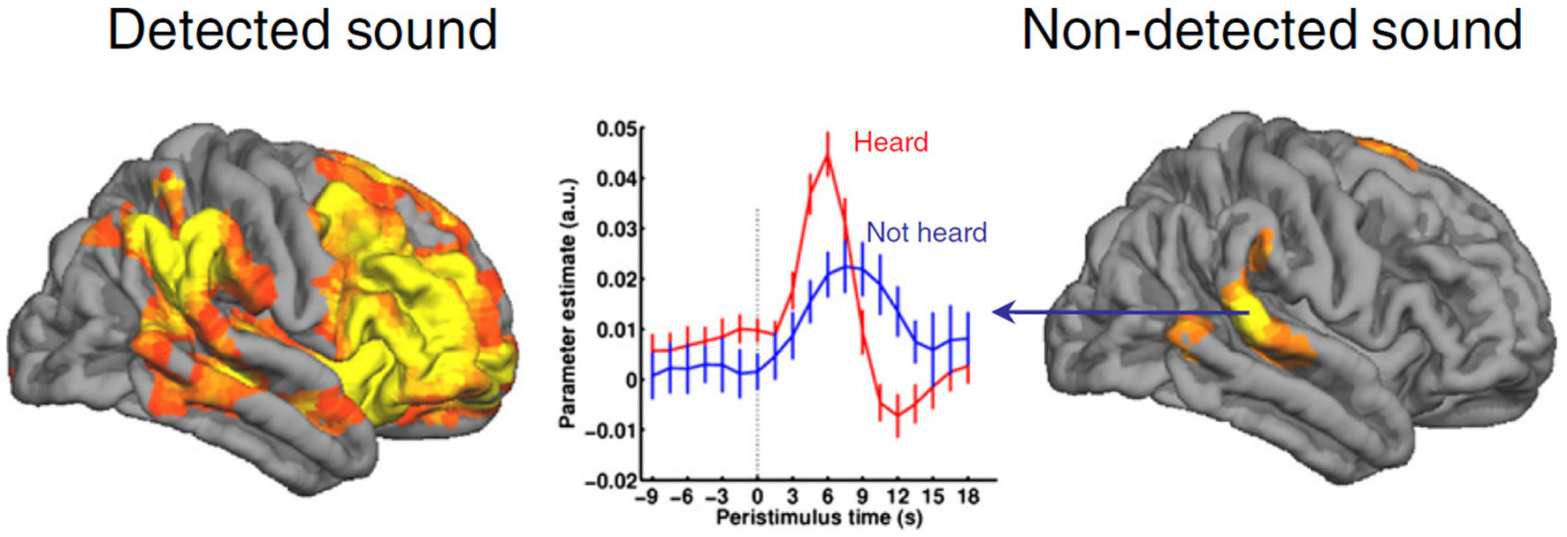
**Consciousness is not correlated with activation of primary sensory cortex**. This example of contrastive analysis demonstrates activation of primary auditory cortex even in the absence of conscious perception. By contrast, detection of the auditory stimulus is correlated with activation of a widespread network prominently involving frontal-parietal networks. Reproduced from Dehaene and Changeux (2011), Neuron, with permission.

A reasonable approach to understanding the mechanism of anesthetic-induced unconsciousness would be to consider the neurobiological underpinnings of conscious experience itself. As noted, consciousness is sometimes reduced to the dissociable processes of wakefulness and awareness. Importantly, wakefulness is neither sufficient nor even necessary for conscious experience. Dreaming is an example of how consciousness can occur in the absence of wakefulness (demonstrating a lack of necessity) and the vegetative state is an example of a presumably unconscious state despite evidence of wakefulness (demonstrating a lack of sufficiency) (Laureys, 2005). In terms of awareness, evidence is accumulating that conscious experience does not correlate with processing at the level of the primary sensory cortex. Numerous studies using contrastive analysis, in which a sensory stimulus is delivered at threshold and then brain activation patterns are “contrasted,” suggest that primary sensory processing is not sufficient for conscious perception of a stimulus (for review see Dehaene and Changeux, 2011). Consciousness is, however, associated with widespread activation of multimodal cortical networks, including frontal, posterior parietal, and temporal areas (Figure 1). To summarize, neither arousal from subcortical structures nor activity of primary sensory cortex is sufficient for consciousness; processing in more distributed networks of association cortex appears to correlate best with conscious experience. It is also worth noting that many current and major theories of consciousness—including global neuronal workspace theory (Dehaene and Changeux, 2011), integrated information theory (Tononi, 2012), predictive coding (Clark, 2013), representationalism (Lau and Rosenthal, 2011)—consider some form of top-down or network-level process to be critical for consciousness.

**Figure 2:**
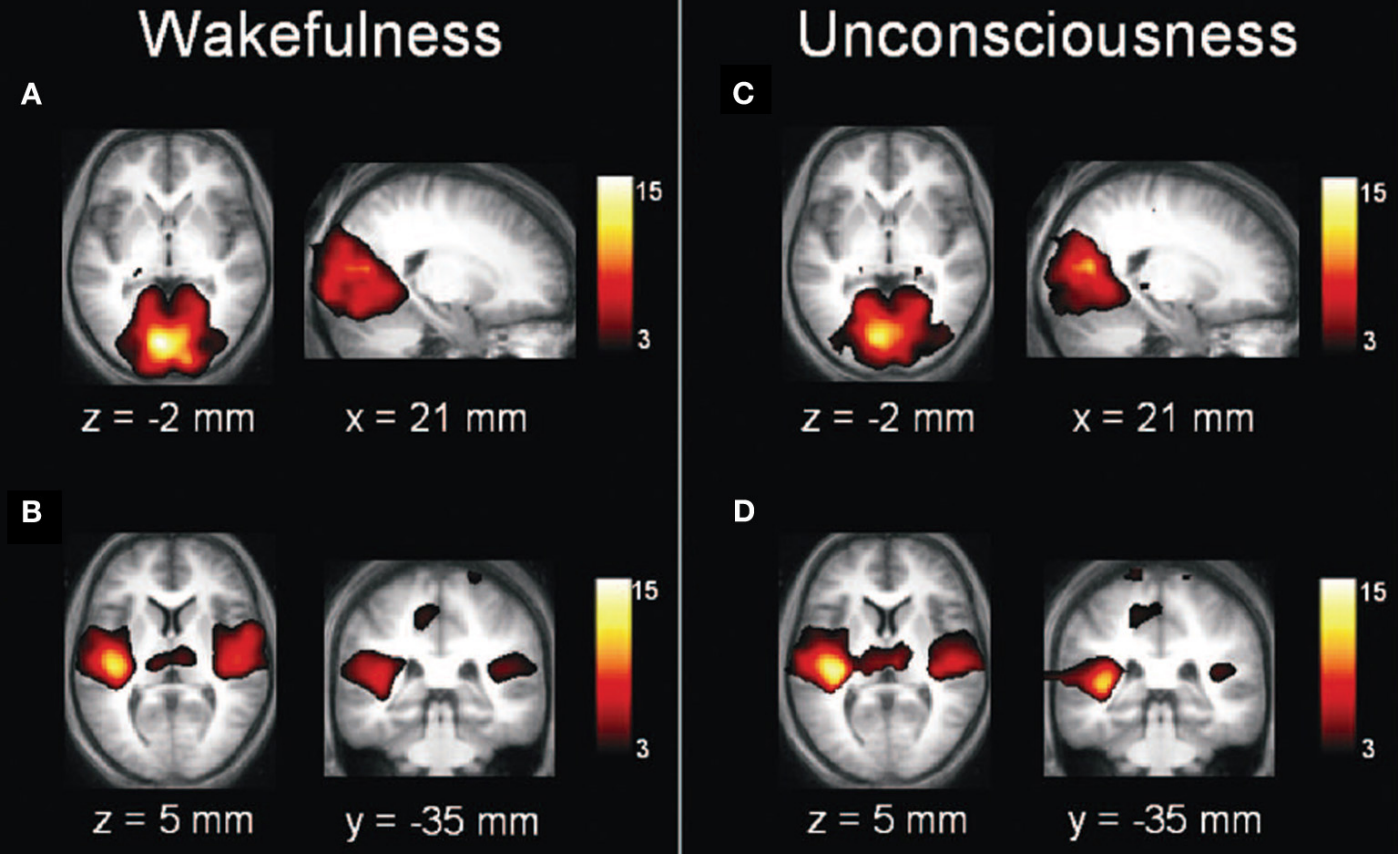
**Anesthetic-induced unconsciousness is not correlated with inactivation of primary sensory cortex**. Transverse and sagittal sections of primary visual **(A,C)** and auditory **(B,D)** cortices during wakefulness **(A,B)** and propofol-induced unconsciousness **(C,D)**; note the relative preservation across states. Reproduced from Boveroux et al. (2010), Anesthesiology, with permission.

Just because consciousness is associated with activation of more extended cortical networks does not necessitate that anesthetic-induced unconsciousness is as well. It is entirely possible that general anesthetics could block the transmission of information from the periphery, inhibit arousal centers, block thalamic relay of information to the primary sensory cortex, or disrupt primary sensory processing. However, neuroimaging data suggest that anesthetic-induced unconsciousness is associated with deactivation of more extended frontal-parietal networks, while primary sensory networks remain relatively intact (Boveroux et al., 2010; Bonhomme et al., 2012) (Figure 2). This is mirrored somewhat in the thalamus by preferential disruption of “non-specific” thalamic nuclei, which are thought to play more of an integrative role for cortical computation rather than a processing station for sensory information (Liu et al., 2013). Again, it is important to note that these findings may relate specifically to anesthetic-induced unconsciousness, the topic of interest, rather than surgical anesthesia. It is clear that higher concentrations of general anesthetics beyond those required for loss of responsiveness in a resting state can suppress primary sensory cortex (Ni Mhuircheartaigh et al., 2013) and at yet higher concentrations can cause more global suppression.

### Consciousness and anesthetic-induced unconsciousness are associated with late evoked potentials rather than early ones

**Figure 3:**
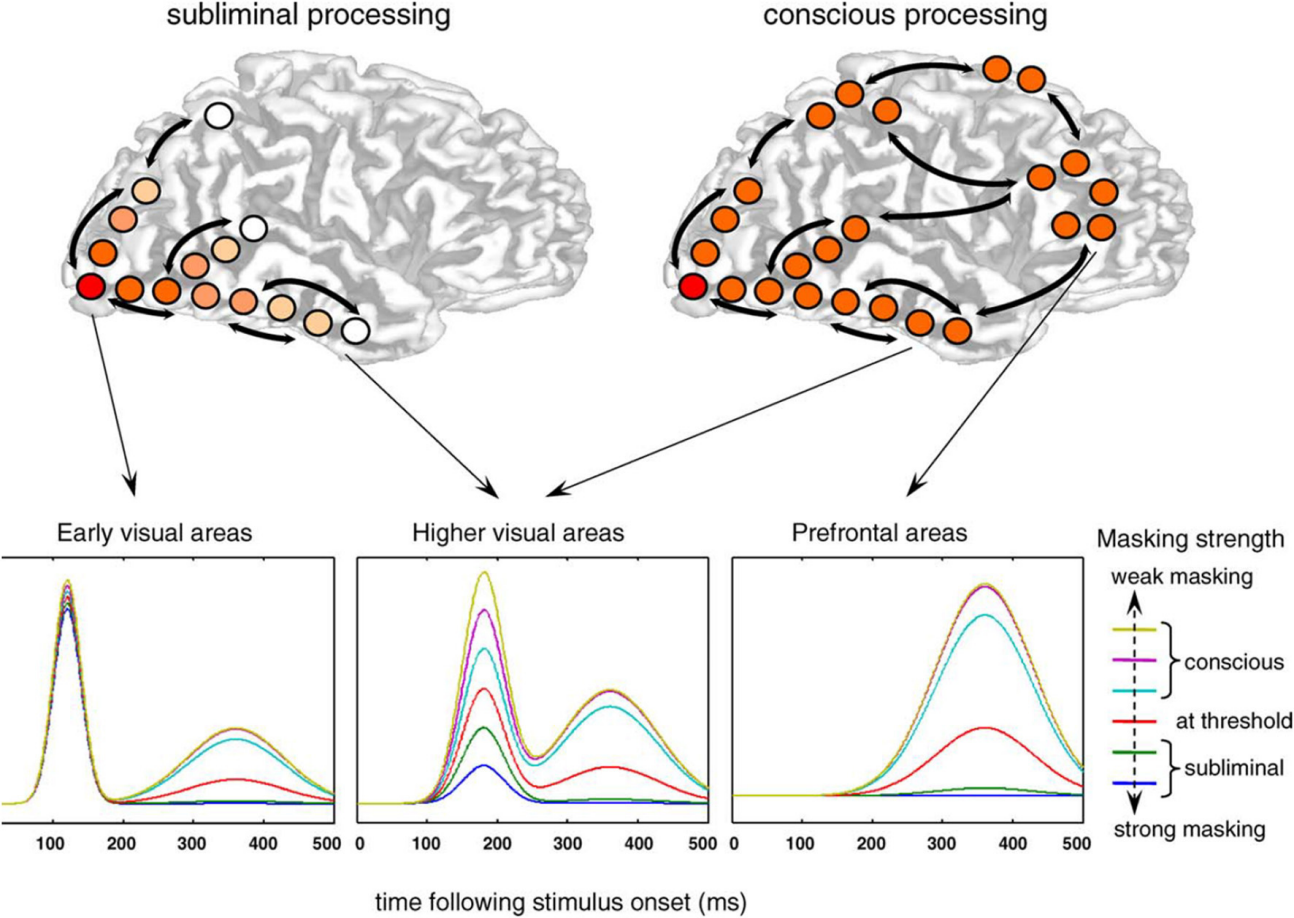
**Consciousness is not correlated with early event-related potentials**. This electrophysiological study of visual processing concluded that early event-related potentials (reflecting more primary sensory processing) are not correlated with conscious perception. Top-down processing from prefrontal cortex was more closely associated with consciousness. Reproduced from Del Cul et al. (2007), PLoS Biology, with permission.

**Figure 4:**
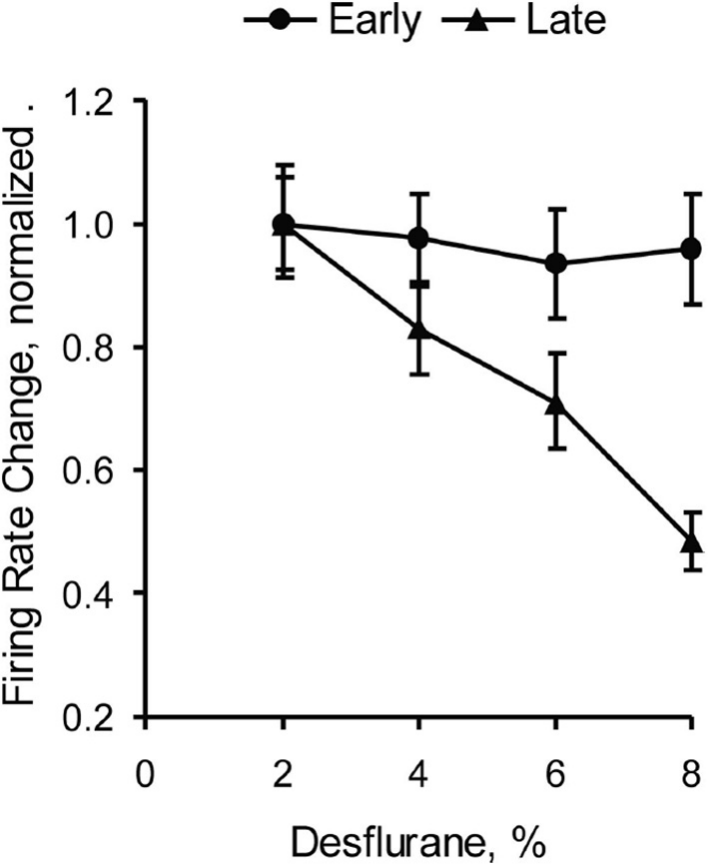
**Anesthetic-induced unconsciousness is not correlated with effects on early evoked potentials**. This study of visual evoked potentials in rats demonstrates a clear dose-dependent effect of the inhaled anesthetic desflurane on long-latency potentials, with sparing of early potentials reflecting processing in primary visual cortex. Reproduced from Hudetz et al. (2009), Anesthesiology, with permission.

The neuroanatomical substrates of consciousness and anesthetic-induced unconsciousness give us a sense of the “where” of consciousness or anesthesia but also have implications for the “when” of consciousness or anesthesia. Studies using event-related potentials suggest that conscious experience is correlated with longer-latency potentials rather than early potentials (Del Cul et al., 2007) (Figure 3). Not surprisingly, evoked-potential data mirror the neuroanatomical structures of interest: early potentials reflect more primary processing, while later potentials reflect more integrative activity beyond the primary sensory cortex (e.g., V1) or higher-order modality-specific processing areas (e.g., V2 and beyond). Likewise, studies of anesthetic-induced unconsciousness have revealed that longer-latency visual evoked potentials are suppressed in a clear dose-dependent manner, while early potentials remain unperturbed (Hudetz et al., 2009) (Figure 4). These longer-latency potentials likely reflect, in part, reentrant processing from anterior to posterior structures, which appear to be preferentially suppressed in association with anesthetic-induced unconsciousness in rats exposed to visual flash stimuli (Imas et al., 2005). It is difficult to see how anesthetics can act in a bottom-up manner in terms of a sensory hierarchy if visual information is still able to be transmitted through the thalamus to the primary cortex and forward through the dorsal stream, with only reentrant processing in the anterior-to-posterior (i.e., top-down) direction affected.

The observation that late cortical potentials are preferentially inhibited by general anesthetics is routine in the clinical practice of neuroanesthesiology, the subspecialty of anesthesiology that focuses on the perioperative care of neurosurgical patients. During both intracranial and spinal procedures, the use of sensory-evoked potentials to monitor neural function is common. In the case of somatosensory evoked potentials—reflecting a pathway from the peripheral stimulation source, to synapses in the medulla, thalamus, primary somatosensory cortex, and multimodal cortex—there is a clear dose-dependent reduction of amplitude and increase of latency. Importantly, late potentials are the first to be suppressed, while subcortically-derived and primary-sensory-related potentials are more robust (Banoub et al., 2003). The preferential susceptibility of late potentials to the effects of general anesthetics is especially evident when considering brainstem auditory evoked potentials. This complex, polysynaptic pathway of early auditory processing is virtually unperturbed by even supratherapeutic concentrations of anesthetics (Manninen et al., 1985). The sensitivity of late cortical potentials—reflecting processing beyond the sensory cortex—to the effects of anesthetics and the remarkable resilience of brainstem potentials make it difficult to argue that bottom-up sensory processes are being disabled, leading to dysfunction of higher-order systems.

### Anesthetic-induced unconsciousness is not dependent on key subcortical sleep-wake nuclei

A variety of anesthetics has been shown to metabolically activate sleep-promoting nuclei and metabolically inhibit wake-promoting nuclei. More recently, the inhaled anesthetic isoflurane has been shown to activate directly the sleep-promoting neurons within ventrolateral preoptic nucleus (VLPO), a key sleep-promoting region in the hypothalamus (Moore et al., 2012). It is remarkable that this general anesthetic—which typically depresses neuronal function—activates neurons that are specifically active during sleep. From the systems-neuroscience perspective, it is not difficult to conceive of how anesthetic-mediated unconsciousness could be a primarily bottom-up process from the perspective of arousal pathways. Sleep is well known to be generated by subcortical structures in the brainstem and diencephalon, with resultant changes in levels of cortical activity and consciousness (Saper et al., 2005). If, indeed, there are shared circuits for sleep and anesthesia—as a number of studies would suggest—then it stands to reason that anesthetic-induced unconsciousness must follow a similar bottom-up path.

**Figure 5:**
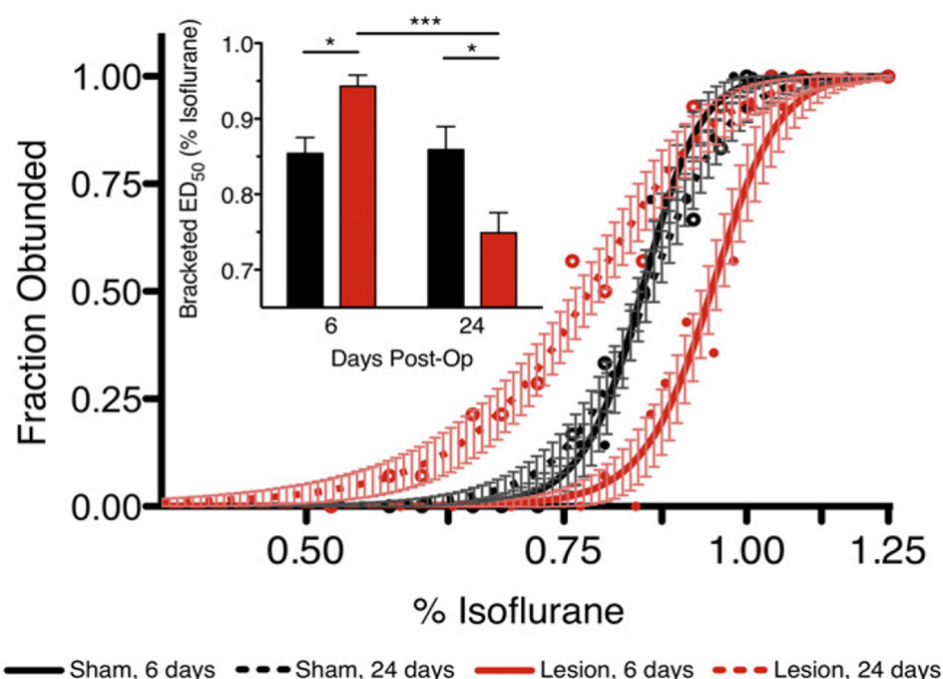
**Ventrolateral preoptic nucleus is not necessary for anesthetic-induced unconsciousness**. Lesions of the ventrolateral preoptic nucleus reveal an acute effect of resistance but a chronic effect of hypersensitivity to the inhaled anesthetic isoflurane. Reproduced from Moore et al. (2012), Current Biology, with permission. ^*^p < 0.05; ^***^p < 0.001.

Although the shared-circuits hypothesis has shown promise, the evidence is far from conclusive. Both intravenous and inhaled anesthetics have been shown to metabolically activate VLPO (Nelson et al., 2002; Moore et al., 2012), which would suggest that VLPO is a key mediator of anesthetic-induced unconsciousness and would lead to the prediction that lesions of the VLPO would eliminate or attenuate the ability of an anesthetic to induce unconsciousness. However, lesions of the VLPO have only an acute effect in conferring partial resistance to inhaled anesthetics. Two studies have demonstrated that, with prolonged insomnia in the absence of a functioning VLPO, animals ultimately become hypersensitive to the effects of anesthetics (Eikermann et al., 2011; Moore et al., 2012) (Figure 5). VLPO is therefore neither sufficient nor necessary for anesthetic-induced unconsciousness. Furthermore, the anesthetic ketamine appears to suppress VLPO activation despite its hypnotic effects (Lu et al., 2008). Collectively, these data suggest that an activated VLPO does not play a critical role in anesthetic-induced unconsciousness.

An alternative bottom-up mechanism of anesthetic-induced unconsciousness that could be mediated through sleep-wake circuitry is the inhibition of subcortical arousal centers by general anesthetics. However, key arousal nuclei have been shown to have a limited role in anesthetic mechanisms. For example, orexinergic neurons in the hypothalamus do not appear to play a role in anesthetic-induced unconsciousness; rather, they modulate the emergence from general anesthesia (Kelz et al., 2008). Histaminergic neurons in the tuberomammillary nucleus (also in the hypothalamus) have been thought to play a role in anesthetic-induced unconsciousness (Luo and Leung, 2011), but recent data bring the behavioral relevance of this nucleus into question (Zecharia et al., 2012). Suppression of brainstem structures such as the locus coeruleus may mediate anesthetic-induced unconsciousness, since mutant mice lacking dopamine-B-hydroxylase (which is required to synthesize norepinephrine in the locus coeruleus) are more sensitive to anesthetics (Hu et al., 2012). However, ketamine appears to *activate* the locus coeruleus in association with its hypnotic effects (Lu et al., 2008) and is dependent, in part, on noradrenergic neurotransmission (Kushikata et al., 2011). Although this list is certainly not exhaustive, it should be clear that inhibition of a number of key arousal nuclei in the brainstem and diencephalon does not appear to be necessary for anesthetic-induced unconsciousness.

The asymmetric role of some arousal nuclei in the process of induction and emergence suggests that the two processes are not mirror images of one another. Recently, a series of elegant studies has provided strong support for the hypothesis that there is a distinct neurobiology of induction and emergence that accounts for the observation of anesthetic hysteresis (Friedman et al., 2010; Joiner et al., 2013). Hysteresis implies that the process of “coming out” of a state is not simply the reverse process of “going in.” In this context hysteresis is manifested as different anesthetic concentrations for induction of and emergence from general anesthesia: higher concentrations are associated with loss of consciousness compared with recovery of consciousness. Why should this be the case? Evidence suggests that there is a barrier to state transitions, termed neural inertia, which may be mediated by sleep systems (Joiner et al., 2013). Neural inertia could explain hysteresis, but it also has implications for the mechanism of anesthetic-induced unconsciousness. Consider: higher concentrations of anesthetic are associated with induction compared to emergence, with sleep networks accounting for the fact that an organism needs less anesthetic to stay anesthetized than to become anesthetized (Friedman et al., 2010). However, this implies that *sleep networks only play a functional role after anesthetic-induced unconsciousness has already occurred*. Therefore, one implication of neural inertia is that sleep-related processes do not play a critical role in the induction of unconsciousness but rather are active during maintenance of unconsciousness.

### Sedative-hypnotic effects of multiple classes of anesthetics can be explained by a top-down but not bottom-up process

In the nineteenth century, an anesthesiologist could walk into an operating room and use (for example) ether, chloroform or nitrous oxide to induce unconsciousness or, at the very least, a state in which the patient had lost “connected consciousness” of the environment (Sanders et al., 2012). We could argue over whether the state induced by ether was precisely the same state as that induced by chloroform, but we would likely agree that the same *functional* outcome had been achieved. In the twenty-first century, an anesthesiologist can walk into an operating room and use (for example) propofol, ketamine, or sevoflurane to induce unconsciousness. It is likely that the state induced by ketamine is quite different than the state induced by propofol, but again a similar functional outcome would be achieved: the patient would not be responding to commands or other environmental stimuli, the patient would be deemed unconscious based on this loss of responsiveness (not universally true, but usually so), and the clinician could therefore start with the business of the day. Although I have framed this in rather practical terms, it is this similar functional outcome that motivates the inclusion of these structurally and pharmacologically diverse drugs in the class of general anesthetics. The relatively fungible nature of these drugs in inducing unconsciousness is also at the core of the anesthetic mechanisms problem: what is the common property that allows us to use these highly diverse anesthetics in a very similar way?

**Figure 6:**
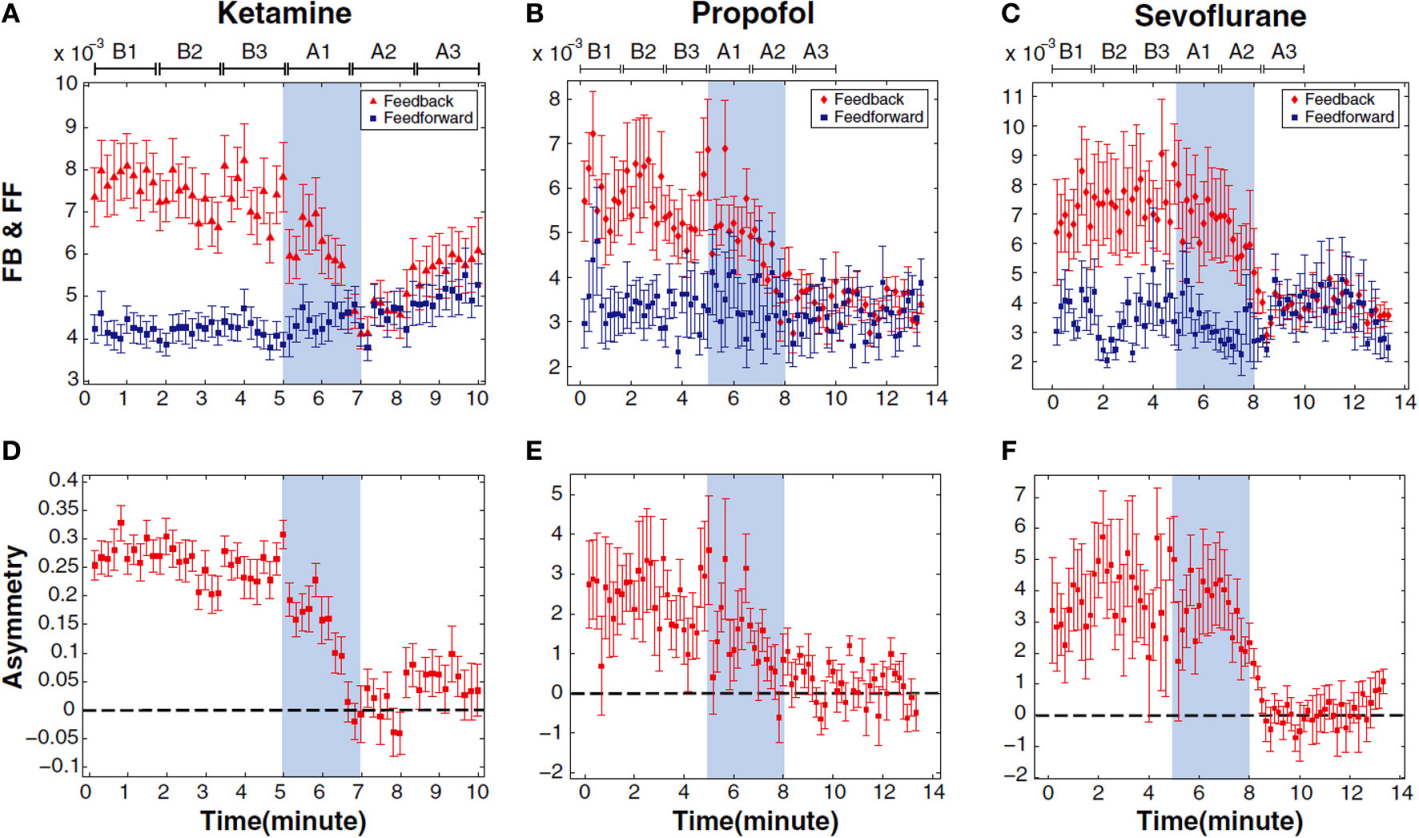
**Inhibition of top-down connectivity is a common correlate of anesthetic-induced unconsciousness across three distinct classes of general anesthetics**. This figure depicts frontal-to-parietal (feedback) and parietal-to-frontal (feedforward) connectivity before, during and after anesthetic-induced unconsciousness in surgical patients **(A–C)**. Lower panels **(D–F)** show asymmetry of directional connectivity, with positive values representing feedback dominance and negative values representing feedforward dominance. Connectivity was measured using electroencephalography and symbolic transfer entropy, which is rooted in information theory. Blue shaded area represents induction of anesthesia; the period before induction is baseline consciousness and the period after is anesthetic-induced unconsciousness. Each state is separated into three substates of Baseline (B1–B3) and Anesthetized (A1–A3) conditions; the timescale is different because patients receiving ketamine were studied using a different protocol than patients receiving propofol and sevoflurane. FB, Feedback; FF, Feedforward. Reproduced from Lee et al. (2013), Anesthesiology, with permission.

It has not yet been possible to identify a single and common bottom-up process that parallels the common functional outcome resulting from the use of propofol, ketamine, and sevoflurane (representatives of the three major classes of general anesthetics). It is ketamine, in particular, that is generally problematic. Unlike propofol, sevoflurane and many other general anesthetics in current use, ketamine fails to (1) act primarily through GABA receptors (Antkowiak, 1999; Salmi et al., 2005; Zhou et al., 2013), (2) activate sleep-promoting nuclei (as noted above) (Lu et al., 2008), (3) metabolically depress the thalamus (Langsjo et al., 2005), or (4) depress fast activity of the electroencephalogram (Lee et al., 2013). In other words, ketamine [and, to some extent, nitrous oxide (Jevtovic-Todorovic et al., 1998)] fails to conform to virtually all bottom-up frameworks of anesthetic mechanisms: molecular, neuroanatomical, systems neuroscience, and even the relatively macroscopic neurophysiologic approach. By contrast, the top-down approach to ketamine-induced unconsciousness has successfully identified a common neural correlate of unconsciousness induced by propofol, ketamine, and sevoflurane that could have been predicted based on the neurobiology of consciousness (Lee et al., 2013) (Figure 6). Loss of effective connectivity from the frontal cortex to more posterior cortices has been consistently observed with all three drugs, suggesting inhibition of reentrant processing as a candidate for the common mediator of anesthetic effects on consciousness (Ku et al., 2011; Jordan et al., 2013). Furthermore, this loss of top-down information processing was selective, because feedforward processing (from posterior parietal to frontal area) appeared preserved. These data are supported by earlier animal studies, as well as more recent studies in humans showing a disruption of cortical communication by various anesthetic drugs (Ferrarelli et al., 2010; Casali et al., 2013). Importantly, this approach reveals parallels with pathological states of unconsciousness (Boly et al., 2011). Although loss of reentrant processing (also referred to as feedback, recurrent, reafferent, or reverberant processing) is currently only correlated with anesthetic-induced and pathological unconsciousness, it is has strong mechanistic implications given its proposed role in consciousness itself (Dehaene and Changeux, 2011). By contrast, no single bottom-up correlate or candidate mechanism of anesthetic-induced unconsciousness across all major classes of anesthetics has been identified or empirically supported.

The association of cortical network events with anesthetic-induced unconsciousness may not be compelling because it is possible that they are simply following the mechanistic parade rather than leading it. We know that modulation of sleep-wake neurons can result in dramatic changes of cortical networks, but can modulation of cortical neurons have a widespread effect? The answer appears to be *yes*. It has been demonstrated that activation of a *single* cortical neuron can lead to transitions in global brain states and produce measurable changes in behavior (Brecht et al., 2004; Houweling and Brecht, 2008; Li et al., 2009), supporting the possibility that even small cortical areas affected by general anesthetics can be instrumental in behavioral and brain state transitions.

### Macro causation can supersede micro causation

One straightforward and plausible way of explaining top-down mechanisms of anesthetic-induced unconsciousness would be that the activity or dynamics of the cortical networks mediating awareness are more susceptible to the effects of general anesthetics than subcortical nuclei mediating sleep-wake states. Demonstrating a more significant contribution—or perhaps an earlier contribution—to unconsciousness by disruption of higher-order cortical processes vs. subcortical or lower-order cortical processes would help resolve the question of top-down vs. bottom-up mechanisms within a sensory or arousal hierarchy. A robust quantitative theory of cortical dynamics will be critical for this line of investigation. However, one might still make the reasonable claim that cortical disruption is not really the true mechanism because it can still be reduced to the underlying molecular mechanisms of anesthetic binding to neurotransmitter receptors or ion channels (i.e., some bottom-up event on a structural scale). It has previously been suggested that it might be helpful to divide anesthetic mechanisms into root and proximate causes, where diverse root causes at the molecular level might be translated across scales to a common proximate cause at the level of cortical information processing (Mashour, 2013). This would lead to a bottom-up framework with individual micro-causes converging on a single macro-level causality. From this perspective, the macro cause would simply supervene on the micro cause. In other words, once the micro level is fixed (e.g., binding of propofol to the GABA_A_ receptor), so too is the macro level—and therefore the underlying micro event has done all of the real causal work.

Recent theoretical efforts have shown that macro-level processes might trump micro-level processes when it comes to true causation. It has been demonstrated in a simulation that a true emergent event can occur in which the macro-level causation contains more effective information than the micro-level causation (Hoel et al., 2013). In other words, *the macro level is actually doing more of the causal work than the micro level*. This suggests the possibility of macro-level *supersedence* in addition to the usual assumption of macro-level *supervenience*. Applying this principle to anesthetic-induced unconsciousness, it is therefore possible that observations at the top-down/macro-level may contain more effective information than the micro-level and thus causally supersede molecular events such as anesthetic binding. This would not imply that anesthetic binding is irrelevant to anesthetic-induced unconsciousness, but rather that it might not add further information to a macro-causal explanation. It is important to note that there is currently no evidence that this is the case. However, it is now at least conceivable that there could be a single causal explanation of anesthetic-induced unconsciousness at the macro level (e.g., cortical dynamics) that is not enhanced by the addition of micro-level information (e.g., molecular binding).

## Discussion

Traditionally, various bottom-up molecular mechanisms have been proposed to explain anesthetic-induced unconsciousness, including disruption of the lipid bilayer and modulation of protein-based neurotransmitter receptors or ion channels. From the neurocognitive perspective, bottom-up explanations of anesthetic-induced unconsciousness have focused on subcortical nuclei that mediate the sleep-wake cycle or early cortical areas that mediate sensory processing. There is growing evidence, however, that general anesthetics disrupt higher-order cognitive processes and that networks of association cortex may be particularly susceptible to anesthetic effects; this evidence parallels current thinking in the science of consciousness. Thus, top-down mechanisms of anesthetic-induced unconsciousness warrant serious consideration. It is possible that the observed changes in the cortex are not simply signatures of lower-order anesthetic actions, but rather that direct modulation of cortical dynamics may be the mechanism of unconsciousness. Furthermore, it is now at least conceivable that such an explanatory framework could represent a sole macro-level causation that supersedes any bottom-up or even molecular description.

Intellectual bias is difficult to escape and thus it is probably better to disclose such bias rather than having it function as a “hidden variable” that shapes the discourse in a covert way. As an anesthesiologist and clinical researcher, my work has focused on the problem of intraoperative awareness with explicit recall and the possibility of brain monitoring techniques that are rooted in the neuroscience of consciousness. As such, the cortex—which is more accessible to study and monitoring in the operating room setting—has been a focus. As a neuroscientist and consciousness researcher, my focus is on the network-level interactions that most current theories deem to be critical for experiential processing. Such clinical and scientific predilections clearly influence my perspective. As such, in this article I have focused on arguments supporting the top-down approach—the reader is encouraged to explore the considerable evidence for alternative perspectives and to draw his or her own conclusions. It is also important to note that bottom-up and top-down perspectives need not be mutually exclusive. This is especially relevant for the higher-order thalamic nuclei, which primarily receive cortical input and are intimately involved in cortical function.

One point deserves emphasis before succumbing to the temptation of disregarding top-down causation on the basis of its inconsistency with the known effects of general anesthetics across species (Crowder, 2008). General anesthetics can stop single-cell organisms, fruit flies, and worms in their tracks… so who needs a cortical network for a satisfactory explanation of anesthetic mechanism? First, it is important to note that the anesthetic endpoint in these model systems is movement alone. Although one could rightfully argue that all endpoints relate to some kind of motor response, the endpoint of impaired movement in *Drosophila* or *C. elegans* cannot necessarily be regarded as a surrogate for impaired perception (van Swinderen, 2006; van Swinderen et al., 1999). It is therefore not even clear that the term “anesthetic-induced unconsciousness” applies here as it would for *Homo sapiens*. Second, as species evolve, so too do the networks governing their behavior and the mechanisms by which behavioral state transitions occur. For example, if we were investigating the mechanism of movement in humans, we might consider the motor cortex and supplementary motor area as starting points. It would be absurd to claim that the motor cortex could not be involved in the primary mechanism of movement in humans based on the arguments that worms move, but worms do not have a motor cortex. Similarly, it would be absurd to claim that higher-order cortical processes cannot be causally central to the mechanisms of anesthetic-induced unconsciousness in humans simply because worms or other species do not exhibit higher-order cortical processes.

## Conclusion

The mechanism of anesthetic-induced unconsciousness is a fundamental question in both anesthesiology and neuroscience, with links to the neurobiology of consciousness itself. An emerging body of evidence suggests that both consciousness (in the experiential sense of the word) and anesthetic-induced unconsciousness are mediated by higher-order processes in the brain. Top-down approaches to anesthetic mechanisms may therefore provide critical insight to both the scientific understanding and clinical practice of anesthesiology.

## Funding

Dr. Mashour is supported by grant RO1 GM098578 from the National Institutes of Health (Bethesda, MD), the James S. McDonnell Foundation (St. Louis, MO), and Cerephex Corporation (Los Altos, CA).

### Conflict of interest statement

Dr. Mashour holds a patent (pending) through the University of Michigan on directional/effective connectivity as a method of assessing consciousness (Application No.: 13/804,706, Filed March 14, 2013, “System and Method to Assess Causal Signaling in the Brain during States of Consciousness”).
